# Psychopathia Sexualis*

**Published:** 1914-01

**Authors:** 


### EDITORIAL DEPARTMENT.

**DR. F. E. DANIEL, Editor** **DR. R. H. L. BIBB, Associate Editor**

DEPARTMENTS { EUGENICS: Drs. M. Duggan and T. Y. Hull.

WOMAN'S DEPARTMENT: Mrs. F. E. Daniel.

### Psychopathia Sexualis.*

"FEW people are conscious of the deep influence exerted by sexual life upon the sentiment, thought and action of man in his social relation to others. Schiller, in his essay, *'Die Weltweisen'* touches upon this subject in these memorable words: 'So long as philosophy keeps together the structure of the Universe, so long does it maintain the world's machinery by hunger and love.' "

From the standpoint of the philosopher, sexual life takes a subordinate position.

*Schopenhauer* (Die Welt als Wille und Vorstellung, 3rd Edition, Vol. 11, p. 586) considers it peculiar that love has hitherto offered material to the poet only, and not also to the philosopher, the scant researches by Plato, Rousseau and Kant always excepted.

Whatever *Schopenhauer* and after him, *E. von Hartmann,* the philosopher of the unknown, discuss about sexual relationship, is so thoroughly incorrect and illogical that, so far as science is concerned, empirical psychology and the metaphysics of man's sexual existence are simply virgin soil. *Michelet's "L'Amour"* and *Mantegazza's* "Physiology of Love" are merely clever causeries and cannot be considered in the light of scientific research.

The poet is the better psychologist, for he is swayed rather by sentiment than by reason, and always treats his subject in a partial fashion. He cannot discern deep shadows, because he is dazed by the blazing light and overcome by the benign heat of the subject. Although the "Physiology of Love," provides inexhaustible material for the poetry of all ages, and of all peoples, nevertheless the poet will not discharge his arduous task adequately without the active cooperation of natural philosophy, and above all, that of medicine, a science which ever seeks to trace all physiological manifestations to their anatomical and physiological sources.

In these efforts medicine succeeds, perhaps, in forming a connection between the pessimistic reflections of the philosopher of the stamp of *Schopenhauer* and *Hartman,** and the gay and naive creations of the poet.

It is not intended to build up in this book a system of the

*Krafft-Ebing's great book. See Book Notices. I have reproduced here, in lieu of an editorial review, the author's preface. It is a much better review than I could give it.—ED.

**Hartman's* philosophical conception of love ("Philosophy of the Unknown," Berlin, 1869) is, "Love causes more pain than pleasure. Pleasure is only an illusion. Reason would demand the avoidance of love, were it not for that fatal sexual instinct. Hence, it would be better to be castrated." *Schopenhauer* expresses the same view in his work: "Die Welt als Vorstellung," 3d edition, Vol. 11, p. 586.

psychology of sexual life, still from the close study of psychology there arise most important psychological facts which it behooves the scientist to notice.

The object of this treatise is merely to record the various psychopathological manifestations of sexual life in man and to reduce them to their lawful condition. This task is by no means an easy one, and the author is well aware of the fact that, despite his (varied) far-reaching experience in psychiatry and criminal medicine, he is yet unable to offer anything but an imperfect system.

The importance of the subject, however, demands scientific research on account of its forensic bearing and its deep influence upon the common weal. The medical barrister only then finds out how sad the lack of our knowledge is, in the domain of sexuality when he is called upon to express an opinion as to the responsibility of the accused whose life, liberty, and honor are at stake. He then begins to appreciate the efforts that have been made to bring light into darkness.

Certain it is that so far as sexual crimes are concerned erroneous ideas prevail, unjust decisions are given, and the law, as well as public opinion are *prima facie* prejudiced against the offender.

The scientific study of the psychopathology of sexual life necessarily deals with the miseries of man and the dark sides of his existence, the shadow of which contorts the sublime image of the deity into horrid caricatures, and leads astray æstheticism and morality.

It is the sad privilege of medicine, and especially that of psychiatry, to ever witness the weakness of human nature and the reverse side of life.

The physician finds, perhaps, a (satisfaction) solace in the fact that he may at times refer those manifestations which offend against our ethical or æsthetical principles to a diseased condition of the mind or the body. He can save the honor of humanity in the forum of morality, and the honor of the individual before the judge and his fellow men. It is from the search of truth that the exalted duties and rights of medical science emanate. * * * *

He appeals to men engaged in serious study in the domains of natural philosophy and medical jurisprudence.

A scientific title has been chosen and technical terms are used throughout the book in order to exclude the lay reader. For the same reason certain portions are written in Latin.

